# Epidermal proteomics demonstrates Elafin as a psoriasis‐specific biomarker and highlights increased anti‐inflammatory activity around psoriatic plaques

**DOI:** 10.1111/jdv.20289

**Published:** 2024-08-19

**Authors:** Anna Berekmeri, Tom Macleod, Isabel Hyde, Gregor Jan Ojak, Caroline Mann, Daniela Kramer, Martin Stacey, Miriam Wittmann

**Affiliations:** ^1^ Leeds Institute of Rheumatic and Musculoskeletal Medicine (LIRMM) University of Leeds Leeds UK; ^2^ National Institute for Health Research (NIHR) Leeds Biomedical Research Centre (BRC) The Leeds Teaching Hospitals Leeds UK; ^3^ Department of Dermatology, University Medical Centre Johannes Gutenberg‐University Mainz Mainz Germany; ^4^ School of Molecular and Cellular Biology University of Leeds Leeds UK

## Abstract

**Background:**

Eczema and psoriasis are common diseases. Despite both showing active epidermal contribution to the inflammatory process, their molecular aetiology and pathological mechanisms are different.

**Objective:**

Further molecular insight into these differences is therefore needed to enable effective future diagnostic and treatment strategies. The majority of our mechanistic and clinical understanding of psoriasis and eczema is derived from RNA, immunohistology and whole skin biopsy data.

**Methods:**

In this study, non‐invasive epidermal sampling of lesional, perilesional and non‐lesional skin from diseased and healthy skin was used to perform an in depth proteomic analysis of epidermal proteins.

**Results:**

Our findings confirmed the psoriasis‐associated cytokine IL‐36γ as an excellent protein biomarker for lesional psoriasis. However, ELISA and ROC curve analysis of 53 psoriasis and 42 eczema derived samples showed that the sensitivity and specificity were outperformed by elastase‐specific protease inhibitor, elafin. Of note, elafin was also found upregulated in non‐lesional psoriatic skin at non‐predilection sites demonstrating inherent differences between the non‐involved skin of healthy and psoriatic individuals. Mass spectrometry and ELISA analysis also demonstrated the upregulation of the anti‐inflammatory molecule IL‐37 in psoriatic perilesional but not lesional skin. The high expression of IL‐37 surrounding psoriatic plaque may contribute to the sharp demarcation of inflammatory morphology changes observed in psoriasis. This finding was also specific for psoriasis and not seen in atopic dermatitis or autoimmune blistering perilesional skin. Our results confirm IL‐36γ and add elafin as robust, hallmark molecules distinguishing psoriasis and eczema‐associated inflammation even in patients under systemic treatment.

**Conclusions:**

Overall, these findings highlight the potential of epidermal non‐invasive sampling and proteomic analysis to increase our diagnostic and pathophysiologic understanding of skin diseases. Moreover, the identification of molecular differences in healthy‐looking skin between patients and healthy controls highlights potential disease susceptibility markers and proteins involved in the initial stages of disease.

## Graphical abstract

Epidermal sampling of lesional, perilesional and non‐lesional skin from psoriasis and eczema patients as well as healthy skin was used to perform an in depth proteomic analysis. IL‐36γ known to be expressed in lesional psoriasis skin was outperformed by the protease inhibitor Elafin as a disease‐specific epidermal biomarker. Elafin is a robust marker presents across psoriasis subtypes and under systemic therapy as long as active lesions are present. Mass spectrometry and ELISA analysis also demonstrated the upregulation of the anti‐inflammatory molecule IL‐37 in psoriatic perilesional but not lesional skin. This anti‐inflammatory activity at lesion border may contribute to the sharp demarcation seen in psoriatic plaques.

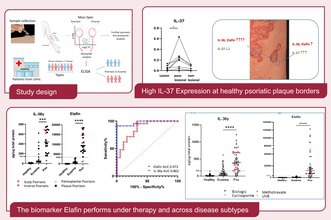

**Why was the study undertaken?**

- This study aimed to identify which epidermal biomarker detectable in non‐invasive sampling shows the best diagnostic performance to distinguish eczema from psoriatic lesions across psoriatic subtypes and under real‐life therapeutic conditions.

**What does this study add?**

- Elafin is a robust psoriasis‐specific biomarker that performs across disease subtypes and in patients under systemic therapy. Epidermal sampling highlights the gradient of the anti‐inflammatory IL‐1 family member IL‐37 which is highest at the healthy plaque border.

**What are the implications of this study for disease understanding and/or clinical care?**

- Overall, these findings highlight the potential of epidermal non‐invasive sampling and proteomic analysis as a diagnostic point‐of‐care test.

## INTRODUCTION

Psoriasis and eczema are common chronic inflammatory skin diseases affecting 2%–3% and up to 10% of the adult population in Western Europe, respectively.^1^, ^2^ Both diseases cause considerable burden to the patient, their families and the health system.^3^

Whilst both diseases usually show typical clinical presentations, there are still areas of diagnostic uncertainty. Children often present with atypical or changing morphology, and very small lesions with minimal disease activity or presentations in the palmoplantar area or the outer ear canal can cause diagnostic problems.^4^ Flexural psoriasis may stay unrecognized, particularly in primary care settings, as it can be confused with fungal or bacterial infection.

There are a number of compelling reasons why an early and correct diagnosis is essential even in cases of minimal disease activity. Firstly, early treatment by disease‐specific highly effective therapies (pathway targeting biologics) can change the course of the disease and may facilitate remission.^5^, ^6^ Appropriate disease specific and early therapy may also help to prevent development of psoriatic co‐morbidities psoriatic arthritis and cardiovascular events. Lastly, early and effective treatment helps to significantly reduce the disease burden for the patients and improves mental health and quality of life.

The current diagnostic gold standard in clinically unclear cases is to assess a skin biopsy by dermatohistopathology. A point‐of‐care, non‐invasive and easy to perform diagnostic approach would therefore be highly desirable to avoid traumatic biopsies and to allow repeated sampling of the same site to monitor disease course. Furthermore, the ability of non‐invasive diagnostics to obtain the molecular signatures from different subtypes will allow targeted therapy towards disease endotypes, which possess distinct pathological mechanisms, despite displaying similar clinical presentations.^7^

To date, there is a large body of literature detailing differentially regulated molecules in psoriasis and eczema. However, the majority focus on selected molecules and compare the lesions of patients with uninvolved skin or to the skin of healthy individuals but not to other inflammatory skin conditions. Recent developments have allowed for more unbiased approaches using RNA microarrays and RNAseq techniques and a range of studies compared disease subgroups or disease entities.^8^, ^9^, ^10^, ^11^, ^12^ This information, however, relies mostly on biopsy samples containing both dermal and epidermal material and mainly analyse mRNA and not protein which ultimately is the key information required to understand disease pathology. Here, we have optimized the technique of tape‐stripping sampling of the epidermis for quantitative mass spectrometry analysis.

This approach allowed us to compare lesional eczema and psoriasis as well as perilesional, healthy‐looking skin in an unbiased proteomic approach. This research is of interest to advance precision medicine where therapeutics are tailored to the underlying inflammatory molecular profile.

## MATERIALS AND METHODS

### Patient recruitment

**FIGURE 1:**
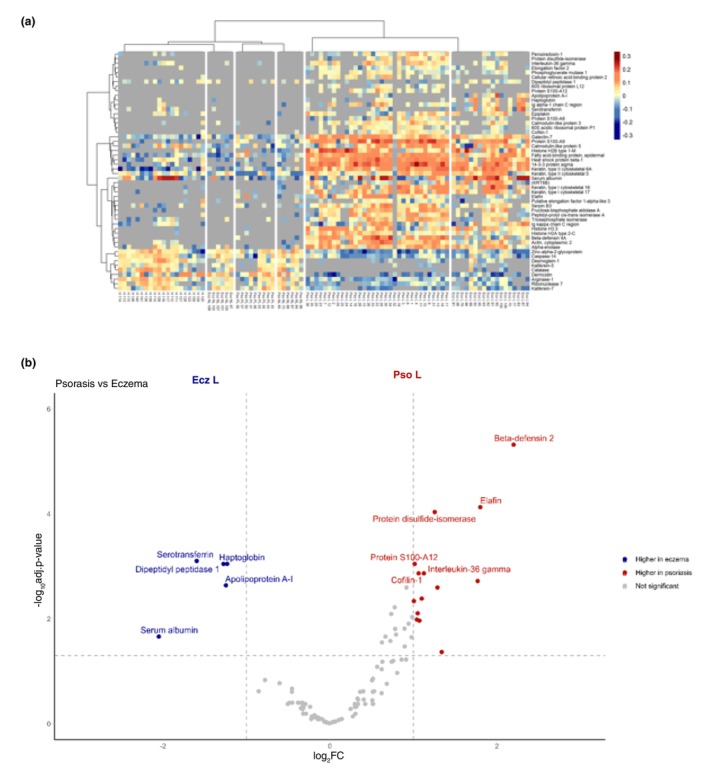
Epidermal sampling demonstrates significantly different proteomic profiles between psoriasis and eczema patients. (a) Heat map of MS results including healthy (H), psoriatic lesional (Pso L), psoriatic peri‐lesional (Pso PL), psoriatic non‐lesional (Pso NL), eczema lesional (Ecz L) and eczema non‐lesional (Ecz NL) skin tape samples. Top differentially expressed proteins from all comparisons are displayed. Abundance values have been normalized to log2 fold change from protein average, and missing values are represented in grey. Hierarchical clustering displayed for proteins and samples, and clustering of samples was performed by group average. (b) Volcano plot analysis depicting the differential expression of proteins between lesional psoriasis (on the right in red) and lesional eczema (on the left in blue). Vertical and horizontal lines denote absolute fold change greater than 1 and FDR adjusted *p* value < 0.05. Top 5 proteins annotated on each side.

**FIGURE 2:**
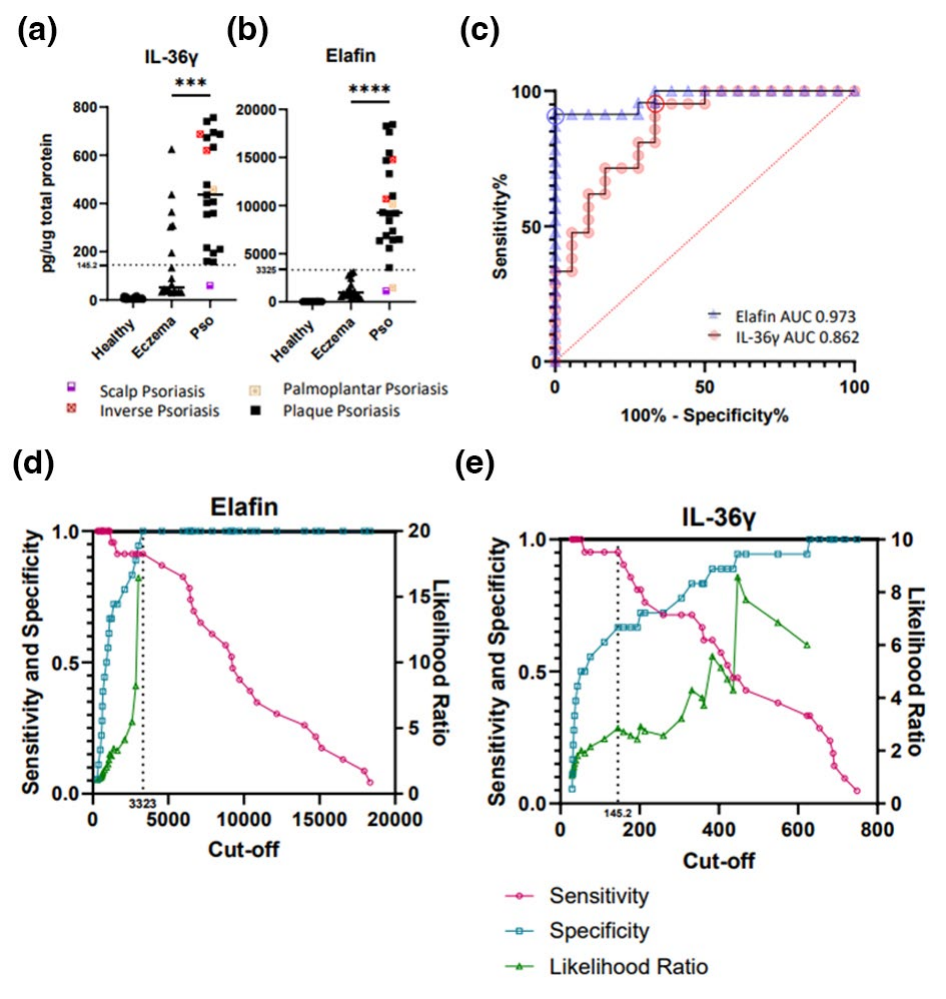
High IL‐36γ and elafin protein expression characterize psoriatic inflammation. ELISA measurement of IL‐36γ (a) and elafin (b) presents in the MS‐matched tape‐strip samples normalized to total protein present in each sample. Subtypes of psoriasis included in the analysis are indicated by colour and shape of the data points as denoted. (c) Response operator characteristic (ROC) curve of the data displayed in a and b to differentiate psoriasis from eczema. Area under the curve (AUC) for each measurement is provided in the graph. Circled points on ROC curve denote optimal threshold defined by Youden index. Threshold protein concentrations are marked by a line on graphs a and b. Cumulative distribution plots show sensitivity, specificity and likelihood ratio values calculated by ROC curve analysis across the range of values measured for elafin (d) and IL‐36γ (e). Statistical significance is denoted by stars; ***p = <0.001, ****p = <0.0001. ns, not significant.

Ethical approval for the recruitment of patients was obtained from the National Research Ethics Service Committees (REC 14/NE/1199, REC 21/SC/0089) and Landesärztekammer Mainz (2022‐16524). The study was conducted in accordance with the declaration of Helsinki. All study subjects have given their written informed consent. Eligible patients over the age of 18, with a clear clinical diagnosis of psoriasis or eczema, were recruited from specialist dermatology clinics from Bradford Teaching Hospitals NHS Foundation Trust and from Leeds Teaching Hospitals NHS Trust, UK. Samples from patients with autoimmune blistering diseases were collected in the outpatient dermatology clinic in Mainz, Germany. The diagnosis was made by specialist dermatologists, based on the characteristic clinical features and /or diagnostic dermatohistopathology. Atopy status was determined by clinical history and/or presence of specific IgE. For mass spectrometry analysis (Figures 1 and 2, Table 1 and Table S1a,b), 11 healthy, 26 psoriasis and 18 eczema patients were recruited mostly not receiving systemic treatment at time of sampling and the sampling site was not treated with topical corticosteroids/calcineurine‐inhibitors/calcipotriol for at least 48 h prior to sampling. A second cohort (Table S2a,b) of 53 psoriasis and 42 eczema patients was recruited in a consecutive manner from routine clinics reflecting ‘real‐world’ situations, where patients with active treatment were also included into the study as long as symptomatic, inflamed lesional skin was present. Healthy volunteers over the age of 18 years with no personal history of any skin diseases were also recruited as controls.

**TABLE 1:** Regulated epidermal proteins in eczema and psoriasis differential protein expression in tape‐stripping samples of healthy versus non‐lesional, para‐lesional and lesional psoriasis, as well as non‐lesional and lesional eczema.

Main function	Protein name	Gene name	Pso NL (vs. healthy)	Pso PL (vs. healthy)	Pso L (vs. healthy)	Ecz NL (vs. healthy)	Ecz L (vs. healthy)
Pro‐inflammatory cytokines	IL36γ	IL36G	~	↑*	↑*	~	~
Anti‐inflammatory cytokines	IL36 receptor antagonist	IL36RN	↑	↑*	~	↑	~
	IL37	IL37	↑	↑*	↓*	~	~
	IL1 receptor antagonist	IL1RN	~	↑*	~	↑	~
S100 family members	S100A7	S100A7	↑	↑*	~	↑	~
	S100A8	S100A8	↑	↑*	↑↑↑*	~	↑
	S100A9	S100A9	↑↑*	↑↑*	↑↑↑↑↑↑*	↑	↑↑↑↑*
	S100A12	S100A12	↑	↑*	↑*	~	~
Serine protease inhibitors	Elafin	PI3	↑	↑↑*	↑↑↑↑*	↑	↑*
	Antileukoproteinase	SLPI	↑	↑	↓*	~	~
	Serpin A12	SerpinA12	↑	↑*	↓*	~	↓
	Serpin B3	SerpinB3	~	↑*	↑↑↑*	↓	↑↑*
	Serpin B4	SerpinB4	↑	↑	↑*	~	~
	Serpin B8	SerpinB8	↑	↑*	↓*	~	~
	Serpin B12	SerpinB12	↑	↑*	↓*	~	↓
	Serine protease inhibitor Kazal‐type 5	SPINK5	↑	↑↑*	↓	↑	~
	Serine protease inhibitor Kazal‐type 9	SPINK9	↑	↑*	↓	~	~
Serine proteases	Kallikrein 5	KLK5	↑	~	↓↓*	↓	↓↓*
	Kallikrein 7	KLK7	~	~	↓↓*	~	↓
Corneodesmosome degradation enzymes/enzymes involved in desquamation	Zinc‐alpha‐2‐glycoprotein	AZGP1	↑	~	↓*	~	↓
	Cathepsin L2	CTSV	↑	↑	↓↓*	~	↓
	Dipeptidyl peptidase 1	CTSC	~	↑	↓*	~	↑
	Cystatin‐M	CST6	~	↑	↓*	~	↓
	Retroviral‐like aspartic protease 1	ASPRV1	~	~	↑*	~	↑
Antimicrobial peptides	Dermcidine	DCD	↑	↑*	↓↓↓*	~	↓↓*
	Gasdermin A	GSDMA	↑	↑	↓*	↑	↓
	WAP four‐disulfide core domain protein 12	WFDC12	↓	↓	↓*	↓↓	↓
	hBD2	DEFB4A	↑	↑*	↑↑↑↑↑*	~	↑↑*
	hBD3	DEFB103A	~	↑*	↑*	↑	↑
	Human neutrophil peptides 1–3	DEFA3;DEFA1	↑	↑	↑↑*	↑	↑
Sweat associated peptides	Prolactin‐inducible protein	PIP	~	~	↓*	~	↓
Lipid metabolism	Fatty acid binding protein 5	FABP5	↑	↑	↑↑↑↑↑*	↑	↑↑↑↑*
	Apolipoprotein A‐I	APOA1	~	↑*	~	~	↑*
	Proactivator polypeptide‐like 1	PSAPL1	~	~	↓*	~	↓
Glycolysis, Neoglucogenesis	Alpha‐enolase	ENO1	~	~	↑↑↑*	~	↑↑↑*
	Fructose‐bisphosphate aldolase A	ALDOA	~	↑*	↑↑↑↑*	↑	↑↑*
	Phosphoglycerate mutase	PGAM	↑	↑*	↑↑*	~	↑*
	Triosephosphate isomerase	TPI1	~	↑	↑↑↑*	~	↑
	Phosphoglycerate kinase 1	PGK1	↑	↑	↑*	~	~
Proapoptotic proteins, intercellular interactions	Galectin7	LGALS7	↑	↑	↑↑↑*	~	↑↑↑*
	Secreted Ly‐6/uPAR‐related protein 1	SLURP1	↓	↓	↓↓*	↓	↓
Signalling pathways	14‐3‐3 protein zeta/delta	YWHAZ	↑	↑*	↑↑*	↑	↑↑*
	14‐3‐3 protein sigma	SFN	~	↑	↑↑↑↑↑*	~	↑↑↑↑↑*
Intracellular signalling, transcription, inflammation and apoptosis	Cyclophilin A	PPIA	↑	↑*	↑↑↑↑*	↑	↑↑*
Neovascularisation	Thymidine phosphorylase	TYMP	↑	↑*	↑*	↑	~
Keratinocyte differentiation markers	Suprabasin	SBSN	↑	↑*	↑*	~	~
	Calmodulin‐like protein 3	CALML3	~	↑*	↑↑*	~	↑*
	Calmodulin‐like protein 5	CALML5	↓	↓	↑*	↓	↑
Profilaggrin/filaggrin processing	Bleomycin hydrolase	BLMH	↓	~	↓*	~	↓↓*
Urea synthesis	Arginase1	ARG1	↓	↓*	↓*	↓	↓↓*

*Note*: Results from MS analysis are summarized. ~ indicates similar expression as to that in healthy, with lower than 0.5 fold difference; arrows indicate more than 0.5 fold increase ↑, or decrease ↓ in the expression of proteins at different stages of inflammation compared with healthy, by each arrow representing approximately 1 (log 2) fold change; * indicate significant difference.

### Epidermal sampling

D‐squame adhesive discs of 3.8 cm^2^ (Cuderm Corporation, Dallas, TX, USA) were used to sample the skin. Ten tapes were collected from the same location and stored at −20°C. Body location sampled was typically upper extremities, neck or upper torso. Lesions from the elbow area were preferentially selected in typical plaque psoriasis, the retro auricular area for scalp psoriasis, submammary region for inverse psoriasis and the antecubital side in typical atopic dermatitis. Lesions with oozing or broken skin were avoided. Non‐lesional samples were collected from the volar forearm of patients. Perilesional samples were taken as shown in Figure 4c to establish a lesion to non‐lesion gradient.

### Sample processing and protein extraction

Frozen tapes were submerged in either 1.5 mL lysis buffer (20 mM Tris pH 7.4, 150 mM NaCl, 1% Triton X‐100, 5 mM EDTA, 1× protease inhibitor cocktail (Roche, Welwyn Garden City, UK)) for protein measurement or 0.75 mL of 6 M GuHCl 150 mM NH_4_HCO_3_ for mass spectrometry analysis. Tubes were left on ice for 30 min prior to sonication (3 cycles of 20 s sonication, 20 s on ice), then centrifuged at 15,000 *g* for 10 min, followed by supernatant collection. Total protein content of tape extracts was measured using the BCA microplate procedure assay kit according to the manufacturer's instructions (Life Technologies Ltd, Paisley, UK).

### Mass spectrometry (MS)

Proteins were denatured, followed by reduction and alkylation steps, and finally by trypsinization, following an in solution tryptic digestion protocol. 1 μg trypsin with high cleavage activity (Thermo Fisher Scientific) was used for each μg of protein. These steps were followed by solid phase extraction, where the final eluent was subsequently dried down in a speed vacuum, reconstituted in 0.1% TFA (20 μL) and stored at −80°C. Liquid chromatography tandem MS (LC–MS–MS) using an Orbitrap system was performed in‐house by the MS Research Facility, University of Leeds.

### MS data acquisition and analysis

MaxQuant proteomics software package and Perseus software version 1.6.1.3 were used to analyse the obtained MS data. The data were cleaned by filtering and removing false hits. This was followed by data normalization using log2(×) transformation. Missing values were imputed from a down‐shifted normal distribution (mean‐1.8, std*0.5). Differential expression between all major groups was assessed using the Limma package in R.

### Protein measurement from tape extract

Specific cytokines and proteins were measured by sandwich ELISA using commercial DuoSet kits (RnD Systems) according to the manufacturer's instructions. IL‐36γ was measured using an ELISA developed in‐house as previously described.^13^ Concentrations were measured as pg/ml and normalized to each sample's total protein content.

### Statistical analysis

A heatmap was generated using the pheatmap package in R. Proteins not present in any group at >50% were eliminated from the analysis as well as samples with <20 reads. Dendrograms were generated with missing values treated as described above. Proteins were selected for heatmap inclusion if they were in the top 20 significantly differentially regulated proteins for any of the comparisons made. A volcano plot was generated using FDR adjusted *p* values comparing psoriasis and eczema lesions.

Protein quantification data were analysed with GraphPad Prism software, version 9.4.1. One‐way analysis of variance (ANOVA) followed by the Tukey multiple comparison test was used to determine statistically significant differences between groups. The Brown‐Forsythe and Welch ANOVA test was used to correct for variance where standard deviations differed between comparison groups. Receiver operating characteristic (ROC) curve was used to analyse and visualize the diagnostic sensitivity and specificity of different protein markers.

## RESULTS

### Epidermal proteomics of healthy versus lesional skin shows a tissue inflammatory response

A total of 690 proteins were detected by LC–MS in epidermal skin samples and processed using MaxQuant software. Following data filtering and normalization, 106 proteins were analysed to determine significant expression levels between disease and control groups. Results are visualized by heatmap (Figure 1a). For a summary of the most regulated proteins of special interest, see Table 1.

As is well described,^14^ when compared to the epidermal proteome of healthy skin, lesional samples from both eczema and psoriasis skin showed an upregulation in proteins generally involved in tissue inflammatory responses such as S100 proteins, protease inhibitors and proteins involved in accelerated proliferation and reduced differentiation.

### Epidermal proteomics of lesional psoriasis and atopic dermatitis shows disease‐specific characteristics

Lesional psoriasis shows a substantial increase in proteins involved in neutrophil infiltration and activity, including numerous proteases and protease inhibitors, and proteins involved in antimicrobial activity. As summarized in Figure 1b, the most prominent proteins seen in lesional eczema as compared to psoriatic epidermis seem to be serum derived and this is in line with the known barrier dysfunctions including on the level of tight junctions^15^ and the spongiotic aspect typically seen in histopathology. MS analysis of the lesional psoriasis and eczema epidermal samples was performed and 19 significantly differentially expressed proteins between psoriasis and eczema were identified, with 5 increased in lesional eczema and 14 were upregulated in lesional psoriasis (Figure 1b).

IL‐36γ, previously shown to be a psoriatic biomarker,^13^, ^14^, ^16^ was amongst the significantly upregulated proteins identified in psoriatic lesional skin when compared to eczema lesions. Interestingly, several proteins were upregulated to a greater extent than IL‐36γ, most notably human beta defensin 2 (hBD2) and elafin, suggesting they may be strong candidates as psoriasis biomarkers.

Results observed in the MS analysis were validated by ELISA. Epidermal lesional psoriasis (including different clinical phenotypes) and eczema (atopic as well as non‐atopic phenotypes) samples taken in duplicate along with the sample for MS were measured for IL‐36γ and elafin. As shown in Figure 2a,b a significantly higher expression of both elafin and IL‐36γ is observed in lesional psoriasis when compared to lesional eczema. Despite showing promise in the volcano plot, ELISA measurement of hBD2 from tape‐stripped samples of a larger cohort of patients exhibited a significant overlap between eczema and psoriasis (Figure S1).

**FIGURE 3:**
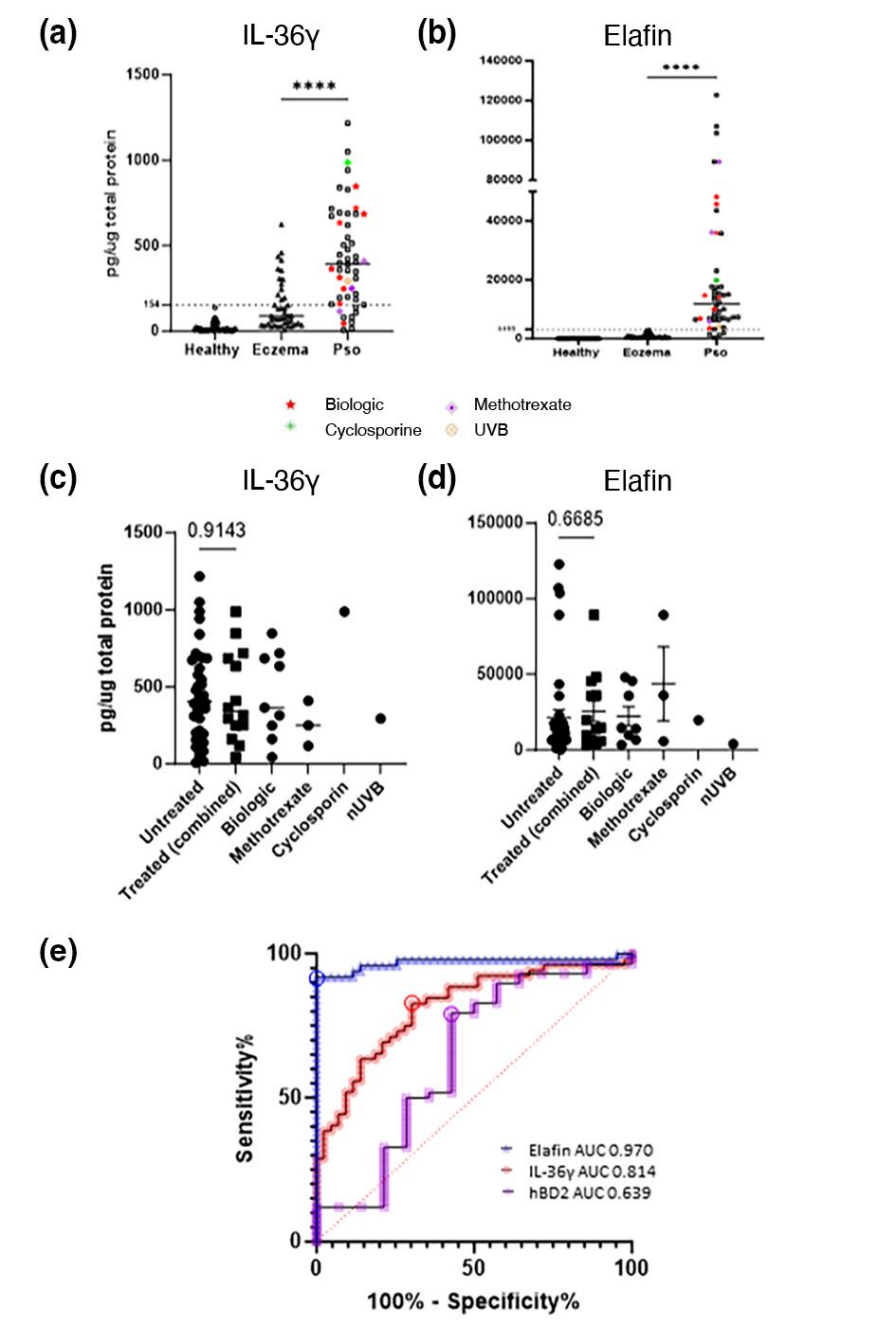
Systemic therapy does not significantly affect expression of lesional IL‐36γ and elafin. ELISA measurements of IL‐36γ (a) and elafin (b) from tape‐strip samples from healthy volunteers, eczema patients and psoriasis patients observed in routine clinics. Measured proteins were normalized to total protein in each tape‐stripped sample. Psoriasis patients are stratified by systemic therapy, as indicated by data point colour and shape denoted in the key below a and b. (c and d) show levels of lesional IL‐36γ and elafin from treated (treatment approaches listed, biologics include TNFi and IL‐17i) and untreated psoriasis patients. (e) ROC curve analysis of eczema and psoriasis patients for IL‐36γ, elafin and hBD2. Area under the curve values are shown in the graph. Circled points on ROC curve denote optimal threshold defined by Youden index. Threshold protein concentrations are marked by a line on graphs a and b. Statistical significance is denoted by stars; ****p = <0.0001. *p* values are displayed where differences are not significant.

We and others have previously demonstrated the diagnostic value of IL‐36γ in distinguishing psoriasis and atopic eczema.^13^, ^14^, ^16^ However, the expression data collected in this cohort including both atopic and non‐atopic eczema as well as other clinical subtypes of psoriasis suggest elafin outperforms IL‐36γ as a biomarker. Indeed, ROC curve analysis generated from the elafin and IL‐36 ELISA data shows convincingly higher specificity for elafin (Figure 2c–e). The diagnostic efficacy of hBD2 was also tested, however, as was indicated by ELISA measurements, ROC curve analysis confirmed hBD2 performed poorly as a diagnostic marker (Figure 3e). Due to the very strong diagnostic value of Elafin (AUC 0.97), a composite test using both elafin and IL‐36γ did not improve the diagnostic power demonstrating that elafin alone is a suitable diagnostic biomarker for psoriasis. Based on the data and thresholds depicted in Figure 3, we calculated the positive predictive value to judge on the diagnostic value of the here described markers. The positive predictive value for Elafin was 100% for Psoriasis (Eczema 91%) and for IL‐36 77% (Eczema also 77%). These calculations however were based on clear clinical diagnoses. We discuss further down overlap presentations and difficult to diagnose morphologies.

### Elafin is a robust marker of psoriatic inflammation

Both elafin and IL‐36ү showed diagnostic value despite the inclusion of diverse clinical subtypes of psoriasis and both atopic and non‐atopic eczema.

Most participants recruited for the MS analysis (Table S1a) were treatment naive or systemic treatment free; however, we were also interested to see if there was diagnostic potential for elafin and IL‐36γ in patients under active systemic treatments reflecting ‘real‐world’ settings. For this analysis, a much larger cohort (Table S2) of active lesional psoriatic epidermal samples was collected from patients representative of those observed in daily clinical practice irrespective of treatment history and were compared with lesional eczema and healthy skin (Figure 3a,b). As shown in Figure 3c,d, conventional systemic therapy and biologics had no significant impact on either biomarker, indicating these markers retain diagnostic efficacy under therapy so long as active lesions are sampled. Furthermore, ROC curve analysis of the larger cohort confirmed efficacy of both elafin and IL‐36γ as sensitive and specific biomarkers and demonstrated the superiority of elafin in this respect (Figure 3e). To determine whether elafin and IL‐36γ were able to act as robust biomarkers within a range of patient types, further analysis was performed investigating age, PASI, disease duration, sex and age of disease onset (Figure S2). There was no strong correlation for either elafin or IL‐36γ with any of these parameters, reinforcing their efficacy as general psoriasis markers across the patient cohort that are not affected by potential confounding factors. However, there was a positive correlation with PASI if the PASI range was limited to a maximum of 15. Interestingly, IL‐36γ correlated with age at disease onset but not with high and low expressor groups.

### Epidermal proteomics demonstrates differences in healthy‐looking skin from patients and non‐diseased individuals

There is evidence that ‘healthy‐looking’ skin in atopic dermatitis and psoriasis is not the same as skin from healthy individuals without a chronic inflammatory skin disease.^17^ Indeed, our proteomics analysis could identify differences in ‘healthy‐looking’ skin between healthy and psoriatic individuals.

**FIGURE 4:**
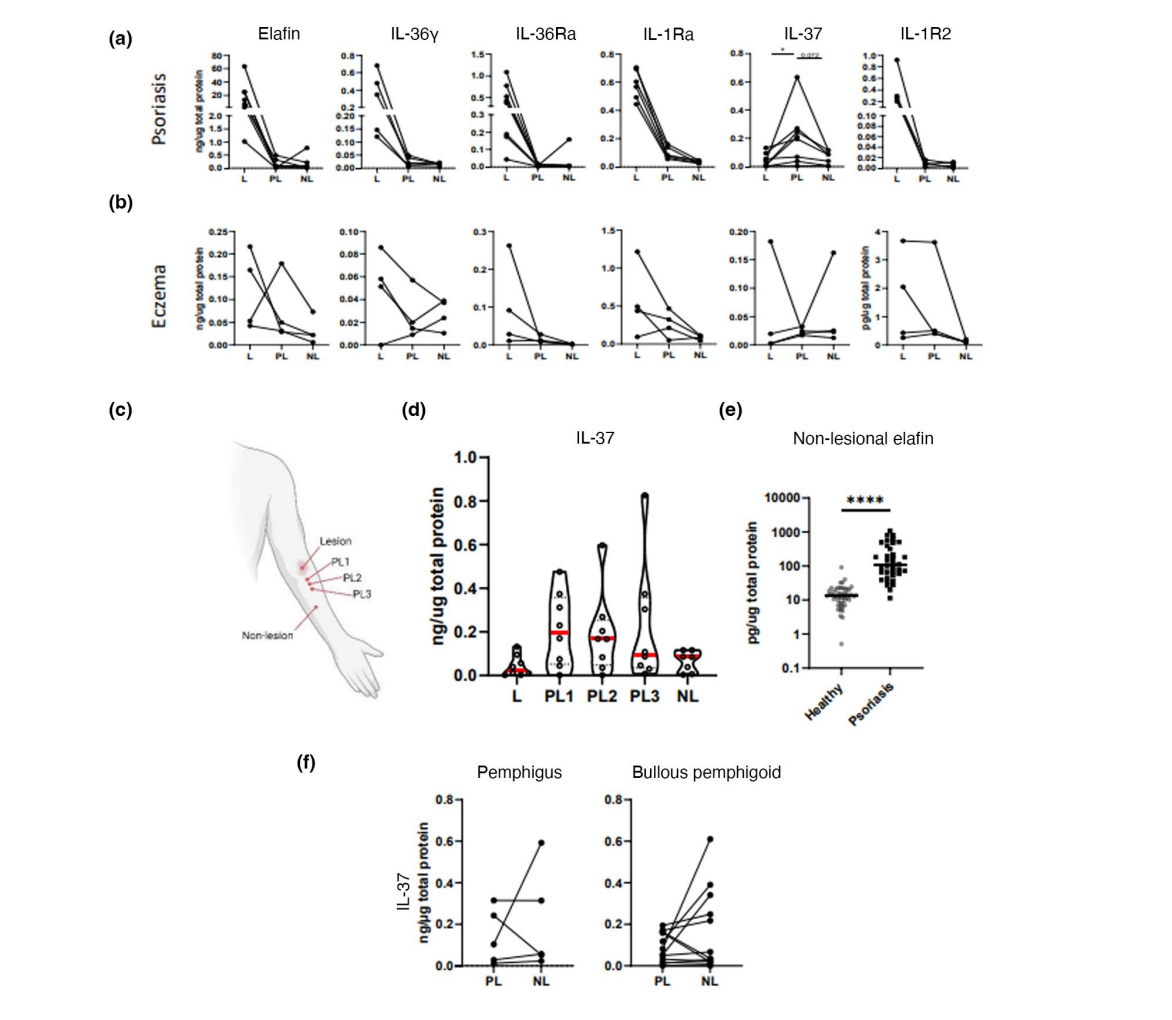
IL‐37 is uniquely regulated across perilesional psoriasis skin. (a and b) show ELISA measurements of elafin, IL‐36γ and the anti‐inflammatory cytokines IL‐36Ra, IL‐1Ra, IL‐37 and IL‐1R2 from donor‐matched lesional (L), perilesional (PL) and non‐lesional (NL) skin tape‐strip samples. Both psoriasis patients (a) and eczema patients (b) were tested. IL‐37 (Panel 5 in a and b) showed a unique pattern of expression in psoriatic perilesional skin so was further measured at increasing distances from the psoriasis lesion, as illustrated in (c) (created using BioRender). IL‐37 was measured from tape‐strip samples adjacent to the lesion (PL1), 3 cm (PL2) and 5 cm (PL3) from the lesion border by ELISA and compared with lesional and non‐lesional tape‐strip samples (d). (e) shows non‐lesional levels of elafin measured by ELISA from tape‐strip samples. (f) Shows results from tape samples taken from non‐lesional and perilesional skin of patients with active autoimmune blistering disease. Measured proteins were normalized to total protein in each tape‐stripped sample. Statistical significance is denoted by stars; *p = <0.05, ****p = <0.0001.

The non‐invasive sampling technique allowed us to sample skin directly proximal to the psoriasis lesion border (perilesional) in addition to non‐lesional skin distant from lesions. MS data indicated perilesional psoriasis skin exhibited differences in anti‐inflammatory cytokines (Table 1). However, ELISA measurement showed that only the anti‐inflammatory cytokine IL‐37 displayed a unique expression pattern in that it was low in lesional but increased significantly in perilesional skin compared with both lesional and non‐lesional skin (Figure 4a). Sequential tape‐stripping adjacent to the psoriasis lesion border (Figure 4c) shows IL‐37 is highly expressed right at the lesion border and trends downwards as the distance from the lesion increases (Figure 4d). A similar pattern of IL‐37 expression was not observed in the lesional, perilesional and non‐lesional skin of eczema or pemphigus vulgaris and bullous pemphigoid patients (Figure 4b,f).

Notably, ELISA measurement supports the fact that epidermal elafin is significantly upregulated in non‐lesional skin from psoriasis patients when compared to healthy controls (Figure 4e).

## DISCUSSION

Although specific molecular signatures have been previously described,^16^, ^18^, ^19^, ^20^, ^21^, ^22^ this work contributes further to the field by focussing on non‐invasive epidermal sampling and proteomics rather than mRNA expression analysis. This is advantageous when envisaging a diagnostic point‐of‐care test, sampling from children and/or repeated testing, multiple samples, for difficult to diagnose flexural, palmoplantar, outer ear canal lesions, for monitoring therapy effects and for primary care professionals inexperienced in diagnosis of skin lesions.

Of the epidermal proteins upregulated in psoriasis lesions compared with eczema, elafin and IL‐36γ were of highest interest. Elafin showed very strong upregulation, whilst IL‐36γ has previously been implicated as a promising biomarker and its emergence in this unbiased approach cements its relevance in this role.^13^, ^14^ Both elafin and IL‐36γ show a link to neutrophilic inflammation. A key effect of activated IL‐36γ on tissue resident cells is the upregulation of IL‐8 which is a potent neutrophil chemoattractant.^23^ Many more functional characteristics of IL‐36γ have been described including its activation of endothelial cells and induction of IL‐23 expression,^24^, ^25^ thus consolidating an IL‐23/IL‐17 dominated inflammatory response. Elafin is a protease inhibitor of neutrophil elastase and is highly expressed by epithelial cells in a subset of respiratory and skin inflammatory responses. Elafin, encoded by the gene *PI3*, has been described to be upregulated in psoriatic inflammation at the mRNA and protein level^26^, ^27^ and has been noted a potential biomarker.^27^, ^28^, ^29^ In this study we show elafin acts as a highly robust protein biomarker from non‐invasive tape‐strip sampling and is also significantly elevated in non‐lesional skin of psoriasis patients when compared to skin of healthy individuals (Figure 4e). Whether elafin in non‐lesional skin contributes to the susceptibility or initiation of psoriasis remains to be investigated.

IL‐36γ is recognized as an epidermal molecule highly upregulated in psoriatic inflammation^14^, ^30^, ^31^ and to a much lesser extent in atopic dermatitis. Despite a broader inclusion criteria of non‐atopic eczema patients, psoriasis subtypes and lesions under treatment IL‐36γ were still able to act as a robust discriminator of eczema and psoriasis.^13^

Both IL‐36γ and elafin show robustness as biomarkers. Most mediators related to inflammatory responses have been shown to rapidly decline in expression under effective therapy.^32^, ^33^, ^34^, ^35^, ^36^ Our analysis, however, has shown that both IL‐36γ and elafin remained unaffected by systemic treatments as long as active lesions are present and sampled.

Although limited in number, the inclusion of less common psoriatic subtypes in the dataset has shown that inverse psoriasis, which can phenotypically resemble eczema or intertrigo, exhibited some of the highest levels of elafin and IL‐36γ measured indicating these markers may be particularly effective confirming the diagnosis of inverse psoriasis and thus represent useful tests in the clinic. Further larger, independent cohorts will be required to fully confirm this observation.

This study focused on well‐defined psoriasis/eczema patients, and this could be a very helpful tool in general practise environments. It is however clear that the real clinical need in dermatology departments is in the area of changing phenotypes, mixed phenotypes and difficult anatomical locations. In particular children may present with different morphology over time causing sometimes uncertainty regarding the underlying diagnosis and predominant inflammatory pathway activated. A non‐invasive, repeatable approach would be very helpful in this scenario, and tape‐stripping method has been published as feasible in very small children.^37^ Changes in morphology are a more recent observation in patients receiving biologics impacting on T‐cell polarization for either psoriasis or eczema. The term flip‐flop patients^38^, ^39^ have been coined for those changing between these diseases under biologics treatment. As this phenomenon is rare we have not yet collected a sufficient number of samples to analyse if this inflammatory pattern shift is correctly reflected in epidermal analysis. Our cohort of true overlap disease is currently small but our preliminary data are in line with publications by Kim et al.^40^ confirming that true overlap scenarios exist with combined mediator expression characteristic for atopic dermatitis and psoriasis. Of interest, the transcriptomic analysis of full thickness biopsies as shown by Kim et al. also includes the markers PI3 (=elafin) and IL‐36. In this context, Bohner et al.^41^ recently published co‐existence of differentially polarized T cells (Type 2 and Type 3) in the same lesion of nummular eczema. With regard to special anatomical location, the palmoplantar area and outer ear canal can cause diagnostic uncertainties when based on morphology alone. The area of molecular markers for subtypes of eczema and psoriasis has clearly advanced in recent years. Our findings are in line with data published by other groups, and tape‐derived epidermal proteomics would certainly be a helpful diagnostic tool in these clinical scenarios.

Our proteomics analysis also highlighted hBD2 as elevated in psoriatic lesions when compared to eczema, yet further investigation demonstrated it to be an unreliable biomarker. A difference in copy number has been described for HBD2 in psoriasis,^42^ and a different expression has long been noted between eczema and psoriasis lesions.^43^ Whilst Type 2 cytokines may downregulate antimicrobial peptides, eczematous lesions often become superinfected with *Staphylococcus aureus* and are still capable of upregulating beta defensins.^44^ Thus, none of the defensins found upregulated in psoriasis serve as solid biomarkers for psoriatic inflammation.

Our data confirm IL‐37 as being uniquely regulated across psoriatic lesional, perilesional and non‐lesional skin. Our results are in line with numerous previous publications reporting decreased expression of IL‐37 from psoriatic lesional biopsies.^45^, ^46^, ^47^ However, our results indicate strong increased expression of IL‐37 around a psoriasis plaque in healthy‐looking skin compared with uninvolved skin sampled from different sites of the body. For atopic dermatitis, transcriptomic analysis has shown a low expression of IL‐37 in lesions compared with non‐lesional skin; however, we failed to observe this in epidermal protein samples.^19^, ^48^

The elevated levels of IL‐37 present at the ‘healthy’ border of psoriatic plaques suggest its involvement in establishing the sharp border between lesion and non‐lesion. IL‐37 might have a functional role in limiting the spread of psoriatic lesions.

Previous work by Ronholt et al.^47^ has demonstrated TNFα and IL‐17 regulate IL‐37 expression in an opposing manner. TNFα increased IL‐37 expression, and the addition of IL‐17 cytokines led to its inhibition. The balance between TNFα, IL‐37 and IL‐17 expression may therefore be important in establishing plaque psoriasis inflammatory borders.

## CONCLUSION

Whilst elafin has previously been noted as a potential biomarker of psoriatic inflammation, this study is the first to demonstrate its significant efficacy in distinguishing psoriasis from eczema in ‘real‐world’ epidermal samples. As the diagnostic efficacy of elafin was not affected by potential confounding factors including, systemic treatment, age, sex, PASI score or disease duration but simply required an active lesion this demonstrates the broad applicability of elafin as a robust biomarker. The combination of such an effective biomarker with a painless non‐invasive sampling technique has great potential in providing a rapid point‐of‐care lateral flow or other diagnostic test which would have significant advantages over invasive and time consuming tissue biopsies and mRNA‐based tests. These findings therefore merit further development as a rapid point of contact diagnostic test and should be considered when facing ambiguous eczematous or psoriatic lesions.

## AUTHOR CONTRIBUTIONS

AB and TM isolated samples and performed the experiments; AB, IH and MS analysed the MS data; AB and MW recruited the patients and summarized patients' characteristics; MW and MS planned and designed the study; GO, CM and DK contributed to critical discussion regarding clinical phenotypes and treatments and helped writing the manuscript. All authors contributed to manuscript writing; AB, TM and IH created the figures. All authors listed contributed intellectually and approved the manuscript for publication.

## FUNDING INFORMATION

This research was supported by a British Psoriasis Association Studentship grant.

## CONFLICT OF INTEREST STATEMENT

The authors declare that the research was conducted in the absence of any commercial or financial relationships that could be construed as a potential conflict of interest.

## Supporting information


Table S1.
Table S2.

Figure S1.

Figure S2.

## Data Availability

The data that support the findings of this study are available from the corresponding author upon reasonable request.
