# Supplementary material for: Epidermal proteomics demonstrates Elafin as a psoriasis‐specific biomarker and highlights increased anti‐inflammatory activity around psoriatic plaques
Source: J Eur Acad Dermatol Venereol. 2024 Aug 19;39(7):1324–35. doi: 10.1111/jdv.20289 (PMC12188514; doi:10.1111/jdv.20289)
Supplement: Supplementary file 1 — Table S1. Table S2. [file JDV-39-1324-s001.docx]

|  | **Psoriasis n=26** | **Eczema n=18** | **Healthy n=11** |
| --- | --- | --- | --- |
|  | **n, %** | **n, %** | **n, %** |
| **Gender** |  |  |  |
| Male | 13 (50.0) | 0 | 5 (45.5) |
| Female | 13 (50.0) | 18 (100) | 6 (55.5) |
| **Age** |  |  |  |
| Mean age | 43.03 | 34.64 | 38.36 |
| 18-47 | 14 (53.8) | 14 (77.8) | 10 (90.9) |
| 48-63 | 9 (34.6) | 4 (22.2) | 1 (9.1) |
| ≥ 64 | 3 (11.5) | 0 | 0 |
| **Topical treatment** |  |  |  |
| NIL | 23 (88.5) | 17 (94.4) |  |
| Applied in the last 48h | 3 (11.5) | 1 (5.6) |  |
| Steroid | 2 (7.7) | 1 (5.6) |  |
| Calcipotriol + Bethametasone | 1 (3.8) | 0 |  |
| **Systemic treatment** |  |  |  |
| NIL | 23 (88.5) | 18 (100) |  |
| Traditional systemic agents | 1 (3.8) | 0 |  |
| Methotrexate | 1 (3.8) |  |  |
| Biologic treatment | 2 | 0 |  |
| Adalimumab (anti TNFα) | 1 (3.8) |  |  |
| Ustekinumab (anti IL12/23) | 1 (3.8) |  |  |
| **Family history** |  |  |  |
| Positive | 15 (57.7) | 8 (44.4) |  |
| Negative | 10 (38.5) | 10 (55.6) |  |
| Unknown | 1 (3.8) | 0 |  |

**Table S1a** **Demographics and clinical characteristics of patient and controls used in the MS analysis.** Topical treatment was marked as zero (NIL) if no topical treatment was used in the last 48 hours.

| **Psoriasis n=26** | |  | **Eczema n=18** | |
| --- | --- | --- | --- | --- |
|  | **n, %** |  |  | **n, %** |
| **Clinical phenotype** |  |  | **Clinical phenotype** |  |
| Plaque psoriasis | 23 (88.5) |  | Atopic | 14 (77.8) |
| Scalp psoriasis | 11 (55.0) |  | Hayfever | 9 (64.3) |
| Palmoplantar psoriasis | 1 (5.0) |  | Asthma | 4 (28.6) |
| Inverse psoriasis | 1 (5.0) |  | Non-atopic | 4 (22.2) |
|  |  |  | Hayfever | 1 (25.0) |
| Inverse psoriasis | 1 (3.8) |  | Asthma | 0 |
| Palmoplantar psoriasis | 2 (7.7) |  |  |  |
| **Disease onset** |  |  | **Disease onset** |  |
| Early onset (< age 40) | 21 (80.8) |  | Childhood onset (< age 18) | 8 (44.4) |
| Childhood onset (< age 18) | 9 (42.9) |  | Adult onset (≥ 18) | 8 (44.4) |
| Late onset (> age 40) | 3 (11.5) |  | Unknown | 2 (11.1) |
| **Psoriatic arthritis** |  |  |  |  |
| Yes | 5 (19.2) |  |  |  |
| No | 13 (50.0) |  |  |  |
| Undiagnosed joint pain | 5 (19.2) |  |  |  |
| Unknown | 3 (11.5) |  |  |  |
| **PASI** |  |  |  |  |
| < 1 | 0 |  |  |  |
| 1-10 | 18 (69.2) |  |  |  |
| >10 | 7 (26.9) |  |  |  |
| Unknown | 1 (3.8) |  |  |  |

**Table S1b** **Clinical characteristics of psoriasis and eczema patients used in the MS analysis.**

|  | **IL36γ/Elafin** | | **hBD2** | |
| --- | --- | --- | --- | --- |
|  | **Psoriasis n=53** | **Eczema n=42** | **Psoriasis n=40** | **Eczema n=17** |
|  | **n, %** | **n, %** | **n, %** | **n, %** |
| **Gender** |  |  |  |  |
| Male | 29 (54.7) | 10 (23.8.) | 18 (45) | 3 (17.6) |
| Female | 24 (45.3) | 32 (76.2) | 22 (55) | 14 (82.4) |
| **Age** |  |  |  |  |
| Mean age | 42.54 | 37.51 | 45.76 | 38.63 |
| 18-47 | 29 (54.7) | 31 (73.8) | 21 (52.5) | 9 (52.9) |
| 48-63 | 19 (35.8) | 11 (26.2) | 15 (37.5) | 8 (47.1) |
| ≥ 64 | 5 (9.4) | 0 | 4 (10) | 0 |
| **Topical treatment** |  |  |  |  |
| NIL | 43 (81.1) | 39 (92.9) | 26 (65.0) | 15 (88.2) |
| Applied in the last 48h | 10 (18.9) | 3 (7.1) | 14 (35.0) | 2 (11.8) |
| Steroid | 3 (5.7) | 3 (7.1) | 3 (7.5) | 2 (11.8) |
| Calcipotriol + Bethametasone | 7 (13.2) | 0 | 10 (25.0) | 0 |
| Salicylic Acid + Bethametasone | 0 | 0 | 1 (2.5) | 0 |
| **Systemic treatment** |  |  |  |  |
| NIL | 37 (69.8) | 42 (100) | 20 (50.0) | 16 (94.1) |
| Doxyxycline | 0 | 0 | 0 | 1 (5.9) |
| Phototherapy | 1 (1.9) | 0 | 2 (5.0) | 0 |
| Traditional systemic agents | 5 (9.4) | 0 | 13 (32.5) | 0 |
| Acitretin | 0 | 0 | 3 (7.5) | 0 |
| Methotrexate | 4 (7.5) | 0 | 8 (20.0) | 0 |
| Ciclosporin | 1 (1.9) | 0 | 1 (2.5) | 0 |
| Fumaric Acid Esters | 0 | 0 | 1 (2.5) | 0 |
| Apremilast (PDE4 inhibitor) | 1 (1.9) | 0 | 0 | 0 |
| Biologic treatment | 9 (17.0) | 0 | 5 (12.5) | 0 |
| Infliximab (anti TNFα) |  |  | 1 (2.5) | 0 |
| Adalimumab (anti TNFα) | 3 (5.7) | 0 |  |  |
| Ustekinumab (anti IL12/23) | 4 (7.5) | 0 | 3 (7.5) | 0 |
| Secukinumab (anti IL17A) | 2 (3.8) | 0 | 1 (2.5) | 0 |
| **Family history** |  |  |  |  |
| Positive | 33 (62.3) | 17 (40.5) | 18 (45.0) | 7 (41.2) |
| Negative | 19 (35.8) | 14 (33.3) | 20 (50.0) | 3 (17.6) |
| Unknown | 1 (1.9) | 11 (26.2) | 2 (5.0) | 7 (41.2) |

**Table S2a Demographics and clinical characteristics of patients used in the “real world” data analysis.** Topical treatment was marked as zero (NIL) if no topical treatment was used in the last 48 hours.

| **Psoriasis** | **IL36γ/Elafin** | **hBD2** |  | **Eczema** | **IL36γ/Elafin** | **hBD2** |
| --- | --- | --- | --- | --- | --- | --- |
|  | **n=53** | **n=40** |  |  | **n=42** | **n=17** |
|  | **n, %** | **n, %** |  |  | **n, %** |  |
| **Clinical phenotype** |  |  |  | **Clinical phenotype** |  |  |
| Plaque psoriasis (PP) | 48 (90.1) | 39 (97.5) |  | Atopic | 30 (71.4) | 13 (76.5) |
| PP+ Scalp psoriasis | 12 (22.6) | 4 (10) |  | Hayfever | 21 (50.0) | 10 (58.5) |
| PP+ Palmoplantar psoriasis | 2 (3.8) | 1 (2.5) |  | Asthma | 12 (28.6) | 5 (29.4) |
| PP+ Inverse psoriasis | 0 |  |  | Non-atopic | 12 (28.6) | 4 (23.5) |
|  |  |  |  | Hayfever | 0 | 0 |
| Inverse psoriasis | 1 (1.9) | 3 (7.5) |  | Asthma | 0 | 0 |
| Palmoplantar psoriasis | 4 (7.5) | 1 (2.5) |  |  |  |  |
| **Disease onset** |  |  |  | **Disease onset** |  |  |
| Early onset (< age 40) | 26 (49.1) | 20 (67.5) |  | Childhood onset (< age 18) | 12 (28.6) | 3 (17.6) |
| Childhood onset (< age 18) | 16 (30.2) | 7 (17.5) |  | Adult onset (≥ 18) | 19 (45.2) | 3 (17.6) |
| Late onset (> age 40) | 11 (20.8) | 7 (17.5) |  | Unknown | 10 (23.8) | 11 (64.7) |
| Unknown | 0.0 | 6 (15.0) |  |  |  |  |
| **Psoriatic arthritis** |  |  |  |  |  |  |
| Yes | 4 (7.5) | 10 (25.0) |  |  |  |  |
| No | 33 (62.3) | 29 (72.5) |  |  |  |  |
| Undiagnosed joint pain | 14 (26.4) | 1 (2.5) |  |  |  |  |
| Unknown | 2 (3.8) |  |  |  |  |  |
| **PASI** |  |  |  |  |  |  |
| < 1 | 4 (7.5) | 1 (2.5) |  |  |  |  |
| 1-10 | 32 (60.4) | 20 (50.0) |  |  |  |  |
| >10 | 15 (28.3) | 15 (37.5) |  |  |  |  |
| Unknown | 2 (3.8) | 4 (10.0) |  |  |  |  |

**Table S2b** **Clinical characteristics of psoriasis and eczema patients used in the *“real world”* data analysis.**
