# Supplementary figures and images for: Epidermal proteomics demonstrates Elafin as a psoriasis‐specific biomarker and highlights increased anti‐inflammatory activity around psoriatic plaques
Source: J Eur Acad Dermatol Venereol. 2024 Aug 19;39(7):1324–35. doi: 10.1111/jdv.20289 (PMC12188514; doi:10.1111/jdv.20289)

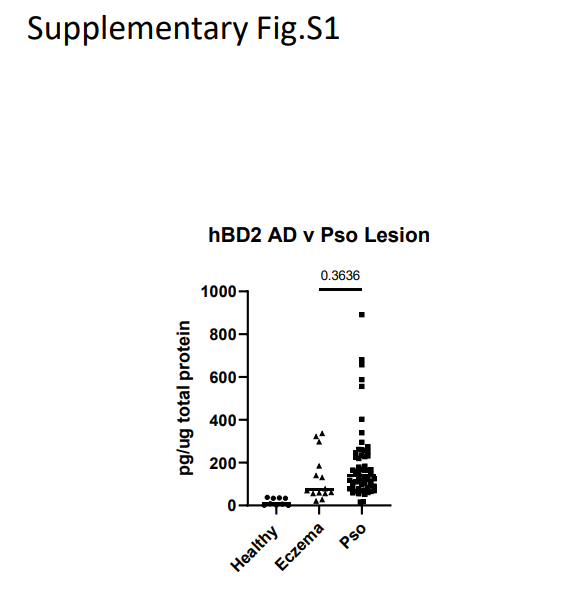

Supplement: Supplementary file 2 — Figure S1. [file JDV-39-1324-s002.png]

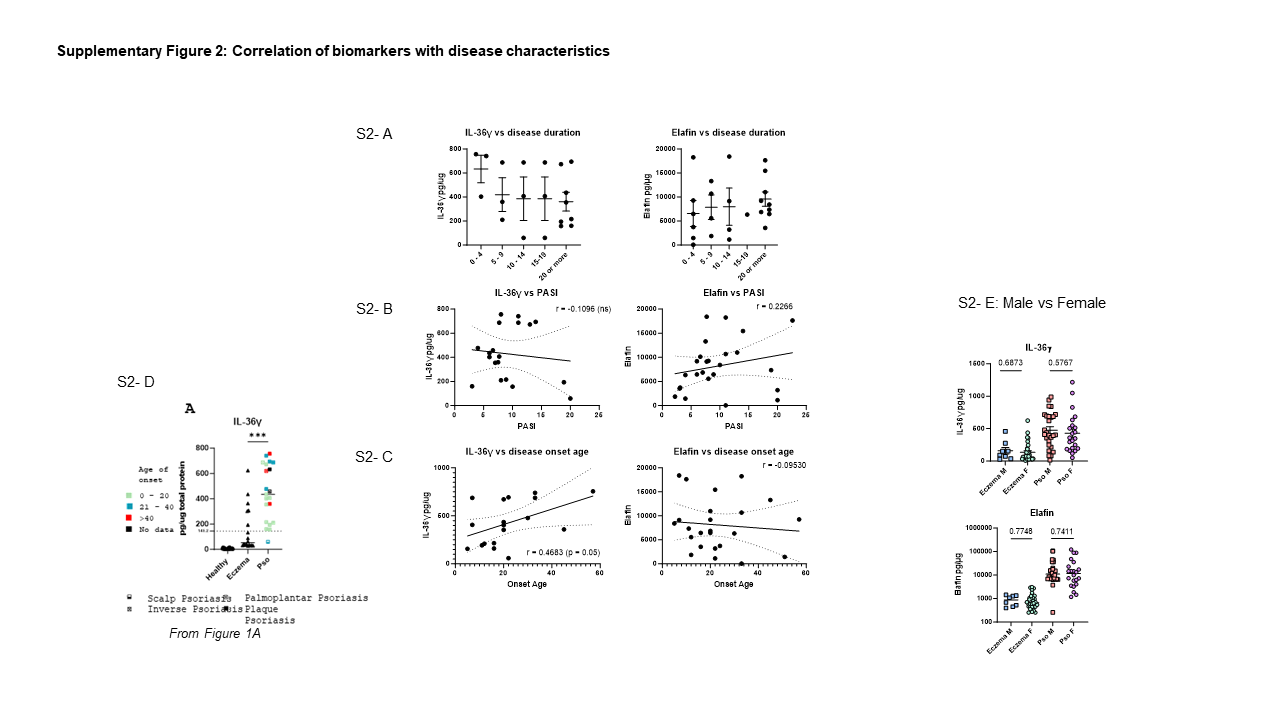

Supplement: Supplementary file 3 — Figure S2. [file JDV-39-1324-s003.png]
